# Modeling the climatic suitability of leishmaniasis vector species in Europe

**DOI:** 10.1038/s41598-017-13822-1

**Published:** 2017-10-17

**Authors:** Lisa K. Koch, Judith Kochmann, Sven Klimpel, Sarah Cunze

**Affiliations:** 1 Goethe-University, Institute of Ecology, Evolution and Diversity, Frankfurt/Main, D-60438 Germany; 2 Senckenberg Gesellschaft für Naturforschung, Senckenberg Biodiversity and Climate Research Centre, Frankfurt/Main, D-60325 Germany

## Abstract

Climate change will affect the geographical distribution of many species in the future. Phlebotomine sandflies are vector species for leishmaniasis, a tropical neglected disease. We applied an ensemble forecasting niche modeling approach to project future changes in climatic suitability for ten vector competent sandfly species in Europe. Whereas the main area of sandfly distribution currently lies in the Mediterranean region, models generally projected a northwards expansion of areas with suitable climatic conditions for most species (*P*. *alexandri*, *P*. *neglectus*, *P*. *papatasi*, *P*. *perfiliewi*, *P*. *tobbi*) in the future. The range of distribution for only two species (*P*. *ariasi*, *P*. *mascittii*) was projected to decline in the future. According to our results, a higher number of vector competent species in Central Europe can generally be expected, assuming no limitations to dispersal. We recommend monitoring for the establishment of vector species, especially in areas with projected climatic suitability for multiple vector species, as a precautious strategy. An increased number of vector species, or a higher abundance of a single species, might result in a higher transmission risk of leishmaniasis, provided that the pathogens follow the projected range shifts.

## Introduction

The subfamily Phlebotominae (sandflies) includes roughly 800 species^1^. Whereas species of the genus *Lutzomyia* occur in the New World, species of the genera *Sergentomyia* and *Phlebotomus* are known to occur only in the Old World^2–4^. Generally, sandflies live in warmer regions, especially in tropical and subtropical regions between 50°N and 40°S^4^. Many sandfly species are established in Europe^5–8^ and their main distribution area can be found in the Mediterranean region, e.g. Portugal, Spain, Italy, Croatia and Greece^9,10^.

Sandfly species are small, hematophagous insects and they are able to transmit several viral and bacterial pathogens^3^. Thus, they are prominent vectors for a large number of diseases, e.g. sandfly fever, summer meningitis, vesicular stomatitis, Chandipura virus encephalitis and Carrión’s disease^4,11^. A well-known disease associated with these species is leishmaniasis^6^. Leishmaniasis is caused by intracellular *Leishmania* parasites. Among the diseases caused by parasites, this disease affects the second largest number of people, after malaria^4,12,13^. Leishmaniasis is a neglected tropical disease^14^ and the main risk areas lay within tropical and subtropical regions. However, recent studies have shown a higher virus diversity than initially suspected in the Mediterranean area, including continental Europe, yielding a higher infection risk for people living south and east of the Mediterranean Sea^4,15–22^. The genus *Phlebotomus* is native to Europe^23^, where dogs are considered main reservoir hosts for the *Leishmania* parasites^24–28^. Other possible reservoir hosts are wild rodents^29^.

Sandflies are characterized as thermophilic, requiring high temperatures for their development and survival. For example, *Phlebotomus papatasi* females are not able to survive temperatures below 15 °C for an extended period of time under laboratory conditions^30^. Larvae of *P*. *perniciosus*, the main vector species in the Mediterranean area, immobilize and die when exposed to cold temperatures between 2 °C and 10 °C^9,31^. Temperature is therefore considered an essential factor for the development and survival of different life stages, and thus, it influences the geographical distribution of sandflies^23,32^. Precipitation and moisture also play an important role in the sandflies’ life cycle. In comparison to other arthropod disease-vectors such as mosquitoes, sandflies do not lay their eggs in water and do not have an aquatic life phase. However, sufficient moisture is still important for egg survival^30,31^. On the other hand, heavy rainfall can restrict flight activity, limit resting site availability for adult sandflies and kill immature stages^33^.

Under future climate change, many sandfly species are expected to expand their ranges in Europe^1,6,9,32,34–36^. However, the extent of their spread as a result of climate change also depends on their dispersal ability^37^. Chances of a far-reaching natural dispersal (>300 m)^38^ seem rather low due the reduced flying abilities of sandflies, but might be compensated for by increasing global trade and travel activities, which would introduce the species quickly beyond current ranges.

The risk of establishment of leishmaniasis in Central and Northern Europe depends on a northward shift of both vector and parasite species. A climate change induced expansion of vector competent species would at the same time lead to an expansion of the risk area for leishmaniasis if disease transmitting *Leishmania* parasites were also able to survive and establish in expanding vector populations. *Leishmania infantum* and *Leishmania tropica* are already established in southern European countries^7^. An increase in the introduction of rescued stray dogs, main reservoir hosts of *Leishmania* parasites, from these areas leads to a spread of the parasites further north^27^. Despite several vector sandfly species and parasitic *Leishmania* species already established in Europe, leishmaniasis is still a largely understudied topic in Central and Northern Europe.

The aim of this study was to investigate the climatic suitability under current and future climatic conditions for ten different sandfly species in Europe using ecological niche modeling (ENM). We focused on species that are currently established in Southern Europe^9,10^. Eight of the considered sandfly species (i.e. *P*. *alexandri*, *P*. *ariasi*, *P*. *neglectus*, *P*. *papatasi*, *P*. *perfiliewi*, *P*. *perniciosus*, *P*. *sergenti* and *P*. *tobbi*) are recognized as vector competent species for leishmaniasis, while the vector competence of two species (i.e. *P*. *mascittii* and *P*. *similis*) has not been verified yet but is strongly suspected^27,39^. The species mainly occur in the Mediterranean region. Some, more cold tolerant species (like *P*. *mascittii*, *P*. *perniciosus* and *P*. *ariasi*) have also been found in more moderate climate regions in Central Europe, like northern France, parts of Swiss, Austria, Belgium and Germany^4,7,8,40^.

Although there have been several studies on the distribution of phlebotomine species^28,37,41,42^, to our knowledge, an extensive modeling approach including several sandfly vector species and the latest RCP scenarios is still missing. We applied an ensemble forecasting approach^43^, which is considered to return a more robust estimation of the habitat suitability compared to single algorithms, which have primarily been used in previous studies. More specifically, we created maps presenting changes in climatic suitability to identify species advancing or retreating under future climate change. We also projected the diversity of the vector species under current and future climatic conditions in Europe to identify future regions prone to new establishments of different sandfly species. The occurrence of vector species is regarded as a risk factor for outbreaks of leishmaniasis in temperate areas^7,32^, however, there are several other factors, primarily the distribution of the associated parasites and its developmental requirements, that should be taken into account for future modeling and risk assessments (first attempts e.g. Pigott *et al*. or Fischer *et al*.^35,44^) to inform efforts to help slow down or even prevent the spread of the disease.

## Results

**Figure 1:**
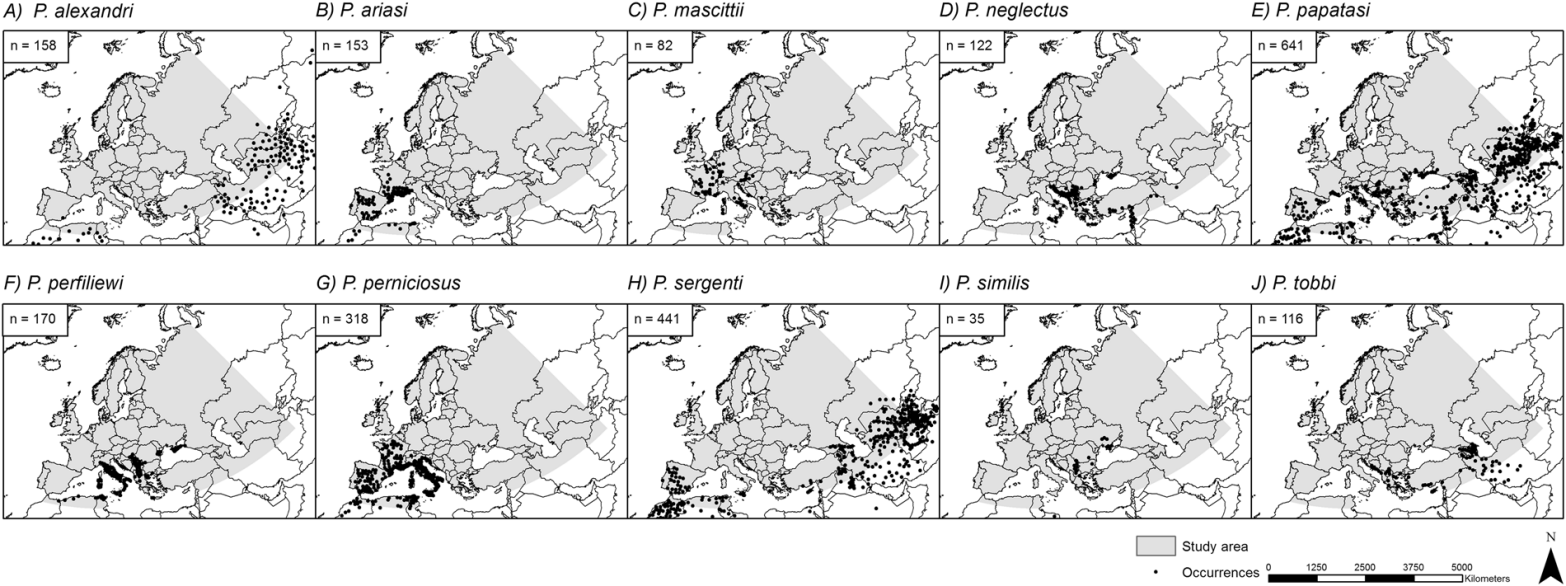
Observed occurrence points of different sandfly species. Original occurrence data points (n) based on literature searches for all ten sandfly species. Note that the number of occurrence points depicted here is bigger than the number of occurrences used in the models (see also Table 1 and Material and Methods). The grey area shows the considered study area. Projected coordinate system: Europe Albers Equal Area Conic. For visualization, maps were built using Esri ArcGIS 10.3^77^ (www.esri.com/software/arcgis).

**Figure 2:**
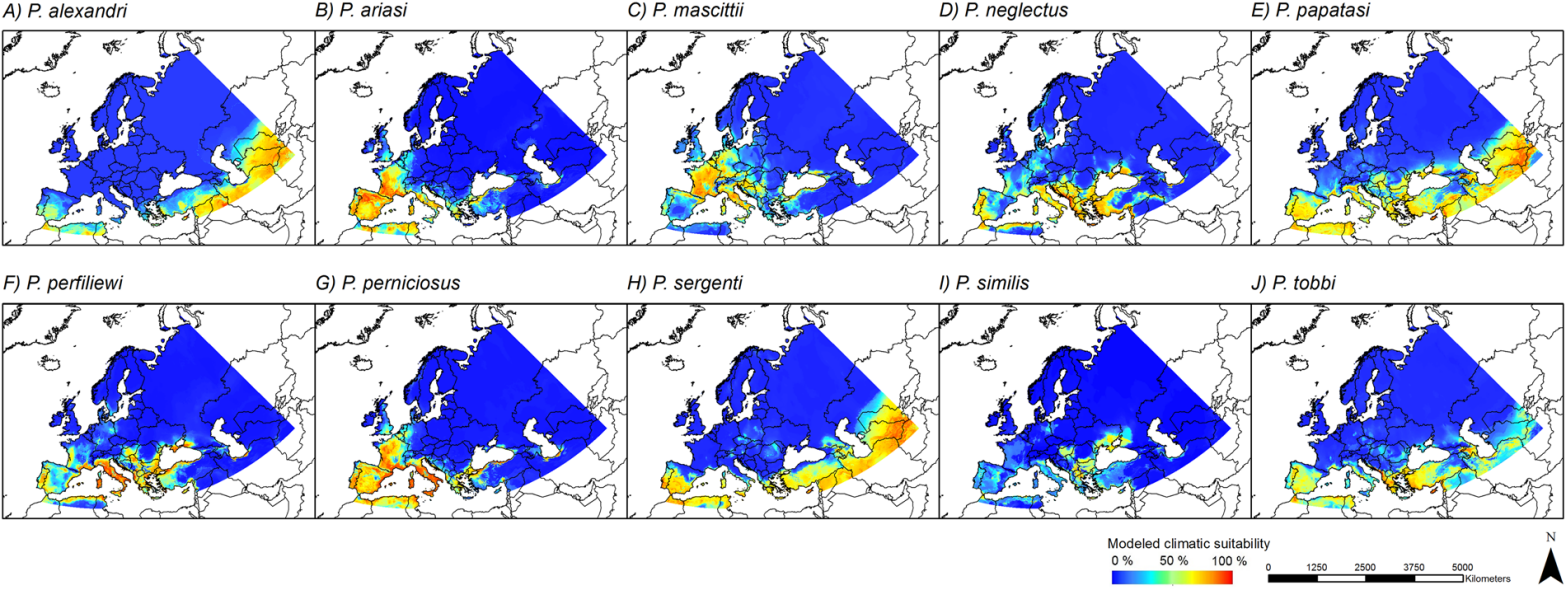
Modeled current climatic suitability. Modeled climatic suitability (consensus model) for all ten sandfly species under current climate conditions. Data was provided by worldclim^67,68^. Projected coordinate system: Europe Albers Equal Area Conic. For visualization, maps were built using Esri ArcGIS 10.3^77^ (www.esri.com/software/arcgis).

The main distribution area for most of the considered species is located in southern Europe. Only a few species, *P*. *ariasi*, *P*. *mascittii* and *P*. *perniciosus*, are distributed in parts of Central Europe (e.g. France) (Fig. 1). The observed distribution is well represented in our modeling results under current climatic conditions (Fig. 2), with AUCs > 0.95 (Supplementary Table S1) in the consensus model for all ten sandfly species. It can be noted that for some species the area of modeled current climatic suitability exceeds the area of observed occurrences. For example, despite projected climatic suitability, *P*. *ariasi* is currently not observed in large parts of Italy, Greece and parts of Turkey.

**Figure 3:**
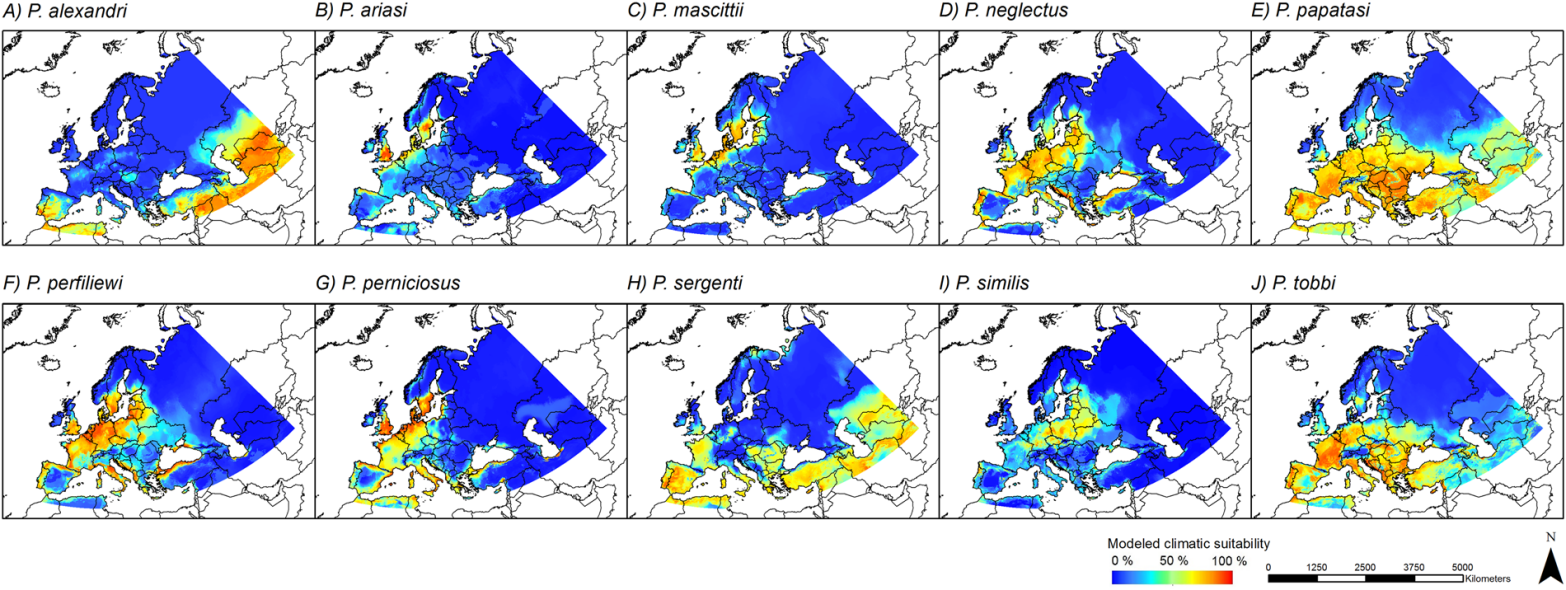
Modeled future climatic suitability. Modeled climatic suitability (consensus model) for all ten sandfly species under future climate conditions (2061–2080 and RCP 8.5, GCM CSIRO-Mk3.6.0). Projected coordinate system: Europe Albers Equal Area Conic. For visualization, maps were built using Esri ArcGIS 10.3^77^ (www.esri.com/software/arcgis).

**Figure 4:**
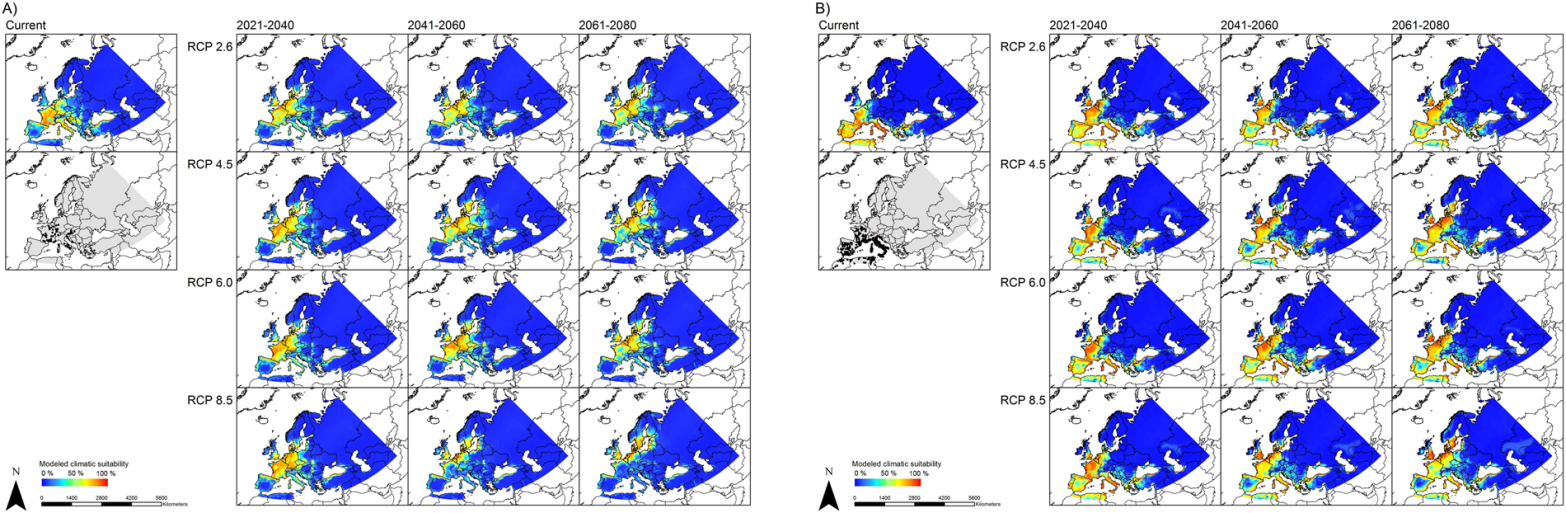
Modeling results for *P*. *mascittii* and *P*. *perniciosus*. Modeled climatic suitability (consensus model) for *Phlebotomus mascittii* (**A**) and *P*. *perniciosus* (**B**) under current and future climate conditions. Data were provided by worldclim^67,68^ (current climate conditions) and the global circulation model CSIRO-Mk3.6.0^72^ (future climate conditions). Projected coordinate system: Europe Albers Equal Area Conic. For visualization, maps were built using Esri ArcGIS 10.3^77^ (www.esri.com/software/arcgis).

Under future climatic conditions (here displayed for the time period 2061–2080, RCP 8.5, Fig. 3), most of the considered species are projected to expand their potential range (becoming climatically suitable in the future) in Central and Northern Europe reaching the island of Great Britain and Scandinavia. A few species, especially *P*. *ariasi* and *P*. *mascittii*, seem to become solely restricted to northern distributions. According to our models, these species will no longer find adequate conditions in their current range of distribution, i.e. in Spain, the Mediterranean area and France, but only further north on the island of Great Britain, in Scandinavia and along the North Sea coast. However, the strong decline of climatically suitable habitats for *P*. *mascittii* can be explained by the strong climatic changes under the scenario RCP 8.5. While the RCP scenarios 2.6, 4.5 and 6.0 predict areas of Central Europe as climatically suited for the species in the future, they clearly differ from the RCP 8.5 scenario (Fig. 4A). Under the RCP 8.5 scenario the climatic conditions in this area are modeled not to be suitable, i.e. they fall outside the species’ niche/requirements (maybe too dry in summer). On the other hand, models for *P*. *perniciosus* predict an increase of climatically suitable areas towards the north over time in the future (Fig. 4B).

**Figure 5:**
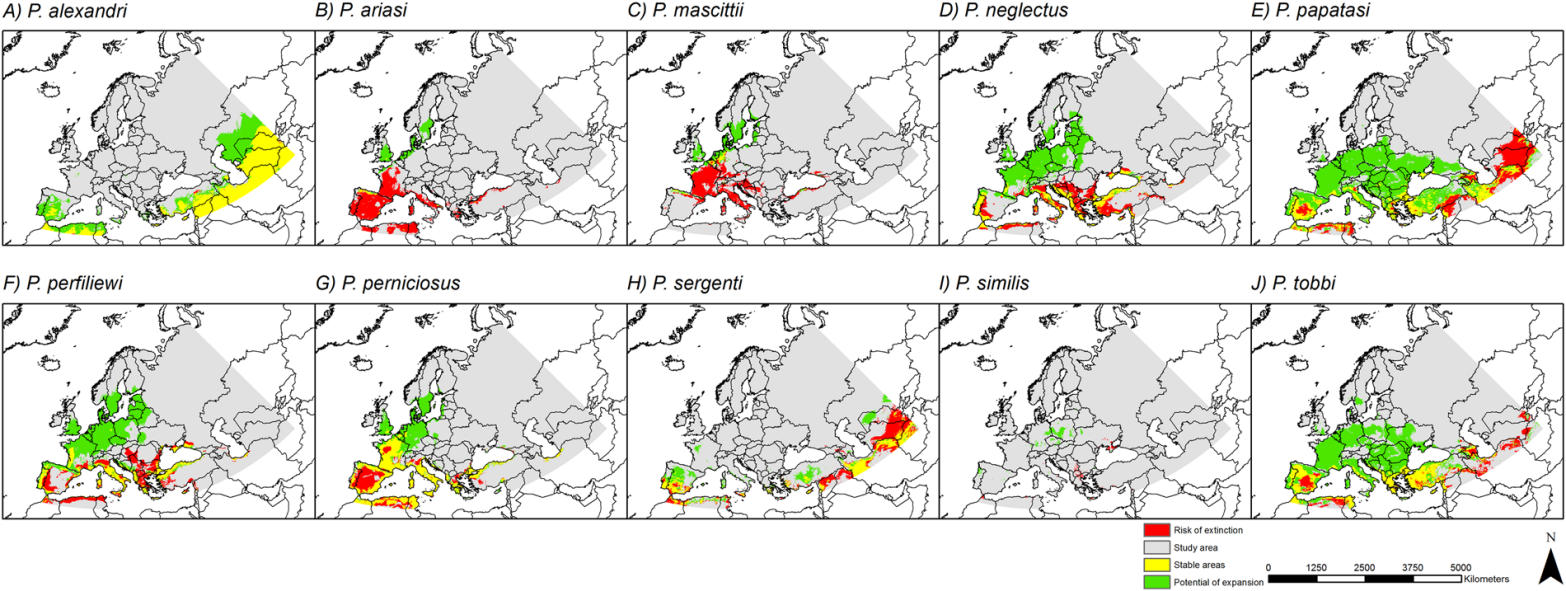
Projected changes in climatic suitability. Comparisons of currently climatically suitable habitats to future climatically suitable habitats (2061–2080; RCP 8.5) based on the binary modeling. Areas with a risk of extinction (red) = areas with suitable climatic conditions only under current conditions; areas with a potential of expansion (green) = areas with suitable climatic conditions only under future conditions; stable areas (yellow) = areas with suitable climatic conditions under current and future conditions. Projected coordinate system: Europe Albers Equal Area Conic. For visualization, maps were built using Esri ArcGIS 10.3^77^ (www.esri.com/software/arcgis).

The aforementioned results are also summarized in the maps showing changes in climatic suitability (Fig. 5), in which the projections of climatic suitability under current conditions are compared to those under future conditions during the time period of 2061–2080 and RCP 8.5. The ten investigated sandfly species can be clearly categorized into: projected expansion (green area larger than red area; *P*. *alexandri*, *P*. *neglectus*, *P*. *papatasi*, *P*. *perfiliewi*, *P*. *tobbi*), decline (red area larger than green area; *P*. *ariasi*, *P*. *mascittii*) and shift (green and red area of roughly the same size; *P*. *perniciosus*, *P*. *sergenti*). For *P*. *similis* the situation is unclear, which may at least partly be explained by the relatively low number of occurrence data (31) and the therefore small areas projected to be climatically suitable under current or future conditions. However, for species like *P*. *alexandri*, *P*. *perniciosus* and *P*. *tobbi*, the climatic conditions are projected to remain suitable over the considered time period (current to 2061–2080) in Southern Europe including southeastern regions as well as the Mediterranean area (indicated in yellow, Fig. 5).

**Figure 6:**
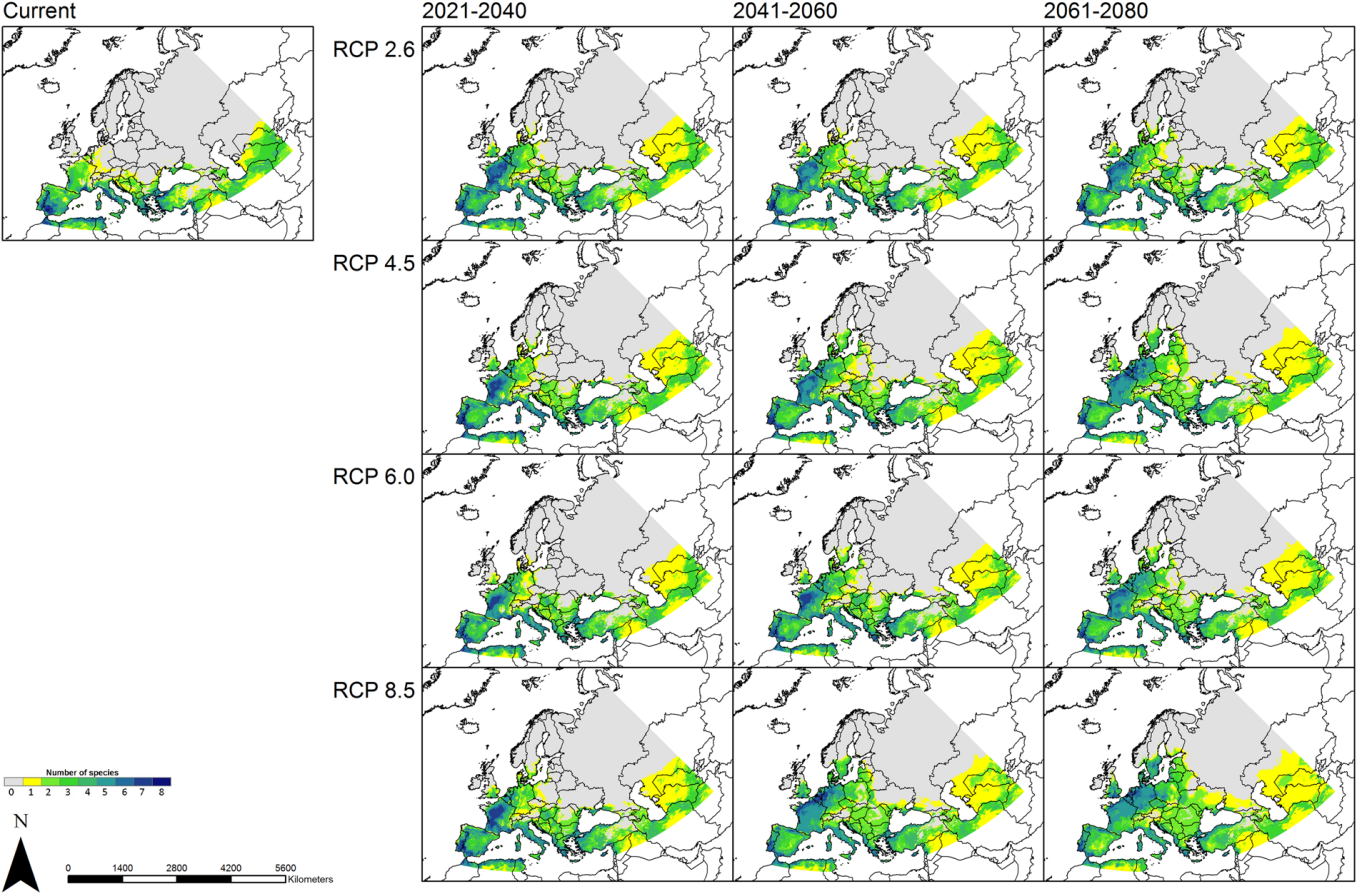
Species diversity. These maps are based on the binary modeling summing up the number of sandfly species across the study area under current and future climate conditions. Projected coordinate system: Europe Albers Equal Area Conic. For visualization, maps were built using Esri ArcGIS 10.3^77^ (www.esri.com/software/arcgis).

The area possessing climatically suitable habitats is projected to expand across the time periods and all RCP scenarios for many of the considered sandfly species. Thus, our generated species diversity maps (Fig. 6) reveal that the number of sandfly species projected to find climatically suitable habitats in Northern and especially Central Europe (northern France, Belgium, the Netherlands, Germany, Denmark and parts of Great Britain and Scandinavia) increases. The number of sandfly species with projected climatic habitat suitability ranges between one and three under current conditions in these areas, and increases up to five species under the RCP 2.6 and up to seven species under the RCP 8.5 (2061–2080).

The most important variable shaping the distribution of the species (all 10 species averaged) in the models (all algorithms averaged) (Supplementary Tables S2–S4) is the mean temperature of the coldest quarter (BIO11), followed by the annual temperature range (BIO7) and the temperature of the warmest quarter (BIO10). The three precipitation predictor variables (BIO15, BIO18 and BIO19) are generally less important for nearly all species than the considered temperature variables. Only for *P*. *alexandri*, precipitation of the warmest quarter (BIO18) is the most important variable.

## Discussion

Climate change is expected to impact species’ potential ranges and thus, also affect species composition in an area. Phlebotomine sandflies, generally characterized as thermophilic species, are assumed to be restricted to areas with warmer climatic conditions, i.e. mainly to the tropics and subtropics. Hence, in Europe, the Mediterranean area is currently assumed to be the only region with suitable climatic conditions where sandflies could potentially occur^1^. However, as a consequence of climate warming, phlebotomine sandflies could expand their potential range towards the north in Europe. Due to their associated health threat for the transmission of leishmaniasis, there is a strong interest in investigating the potential establishment risk of these species. Here, we used ecological niche modeling, a frequently used tool to estimate climate change induced range shifts (e.g. Elith *et al*.^45^), in order to identify areas with climatically suitable habitat conditions for ten different sandfly species in Europe under present-day and future climate. With regard to the number of vector competent species, our work is the most comprehensive study of which we are aware. We included all ten sandfly species that are listed by the ECDC (data from April 2017) as vector competent sandfly species in Europe^9,10^. We considered the RCP scenarios from the fifth Assessment Report^46^, and applied the consensus of 10 modeling algorithms in an ensemble approach. This kind of approach has to our knowledge never been used before for sandfly species. Nevertheless, our results are largely in agreement with other studies using different approaches^41,42^.

In general, models under current climatic conditions largely reflect the observed distribution of the ten investigated sandfly species, with most species occurring in the Mediterranean region. In some areas, however, the models seem to overestimate climatic suitability, i.e. despite predictions of suitable climatic conditions species have not yet been recorded there (e.g. *P*. *ariasi*). This overestimation could be ascribed to different aspects, such as limited dispersal ability, which puts the area out of reach for the species, other factors not related to climatic conditions, or insufficient sampling to detect species’ presence. According to the models underlying a future climate, most of the species, especially *P*. *neglectus*, *P*. *papatasi* and *P*. *tobbi*, are projected to expand their potential area, leading to a projected higher diversity of vector species in Central Europe (especially the Netherlands, Belgium, Germany, Poland and the Czech Republic) in the future, which generally corroborates findings of similar ecological niche models^41,42^. Slight differences can be explained by the fact that we used the latest data on future climatic conditions. Under future conditions, climatically suitable areas for sandflies not only include the Mediterranean area but also regions in Central and Northern Europe, e.g. countries like Austria, Switzerland and Germany, and even regions in Scandinavia and the island of Great Britain. Only a few of the here investigated species show different patterns, namely *P*. *ariasi* and *P*. *mascittii*. Their different patterns might be due to their divergent temperature requirements. *P*. *mascittii* and *P*. *ariasi* are known to predominantly inhabit cooler and humid regions^47^. Similar to the northernmost distributed *P*. *mascittii* ^8,48^, *P*. *ariasi* has not often been recorded in the meso-mediterranean bioclimatic zone (sensu Barón *et al*.^49^: altitude: 600–900 m, annual rainfall: 600–1000mm and mean temperature of 13–17 °C), but instead at higher altitudes in the colder supra-mediterranean bioclimatic zone (sensu Barón *et al*.^49^: altitude: 900–1800 m, annual rainfall: 1000–1600mm and mean temperature of 8–13 °C)^47^. These species might be benefitting less from climate change, which is reflected in the decline of climatically suitable areas under future conditions.

According to our results, winter temperature was found to be the most important variable shaping the distributional patterns of the sandfly species in Europe. These results are consistent with other studies identifying the temperature during winter months as an important factor influencing the diapausing of eggs and the survival of sandflies^9,32^. Temperature seasonality was identified as the second most important variable, showing a gradient from west (low) to east (high), i.e. oceanic vs continental climate, and thus, differentiates the species occurrences in longitudinal direction. The third most important predictor variable according to our models was the temperature of the warmest quarter (BIO10). This is not surprising for thermophilic sandfly species as their distributions correspond to warmer areas that provide temperatures above 15.6 °C for at least several months a year^9^. In contrast to the temperature variables, the three precipitation variables were of lower importance. Although sandflies need a certain amount of moisture for their development and survival^30,31^, heavy rainfalls can kill adults and immature stages^9,33^. In contrast to sandflies, other dipteran species like mosquitoes^50^ have an aquatic life phase and rely on water for breeding. For *Aedes albopictus*, a vector competent mosquito species, summer precipitation was suggested to become a limiting factor reducing climatic suitability for the species in the Mediterranean region in the future^51^. As sandflies lack an aquatic life phase, changes in precipitation patterns projected for the future^9,32^ should therefore be considered less important for their projected ranges.

Ensemble forecasting is based on many algorithms with specific underlying assumptions and data requirements for each algorithm. This method has previously been criticized^52^, however, ensemble forecasting yields a robust estimation of the climatic habitat suitability and is a state-of-the-art approach commonly used to project species’ potential distributions under current and future climatic conditions^43,53^. To obtain reliable results, models generally require that occurrence data reflect the actual distribution of the species (no sampling bias) and that all relevant variables that might influence the spatial distribution of the species are included in the model. With regard to occurrence data, no country-specific monitoring for sandflies exists, therefore, we relied on literature data, but found no obvious data bias (i.e. no explicit pattern of missing occurrences from single countries).

Our models were based on temperature and precipitation variables. However, the distribution of sandflies might also depend on other environmental factors, e.g. soil type, land-use, or wind which can impair their flight activity^38,54^. These variables are typical characteristics of the sandflies’ microhabitats^38,49,55,56^ and are assumed to influence the species’ distribution on a smaller spatial scale^57^. Here, we only used climatic variables that are considered important drivers for large-scale distributional patterns in Europe^23,32,57^.

In addition, biotic interaction (e.g. interspecific competition) is a disregarded factor in ecological niche modeling. This is also an important issue considering the projected sandfly diversity. A higher number of species potentially occurring in a certain area may also lead to higher competition between them. For example, the gain in climatically suitable habitats modeled for *P*. *perniciosus*, which is the main vector species of *Leishmania infantum* in the Mediterranean area^58,59^ could be partly diminished due to the occurrence of outcompeting species. Although *P*. *perniciosus* is found under a broader range of climatic conditions (in two main bioclimatic zones)^47^ and has a high ecological plasticity, potentially enabling the species to adopt faster to changing climatic conditions, the highly specialized *P*. *ariasi* seems to outcompete *P*. *perniciosus*, especially in cooler regions^47^. Furthermore, future projections assume that the species’ niche remains constant over time and space, i.e. the species will not show any physiological adaptations (niche conservatism hypothesis)^60^. However, different lineages with different temperature tolerances already exist, e.g. for *P*. *sergenti* ^23^.

Dispersal limitations were not considered in the models, but might explain why in some areas, the occurrence of sandfly species was not recorded despite models projecting climatic suitability under current conditions (see discussion on overestimation of areas above). A common assumption was that sandflies would be limited in their dispersal ability due to their weak flying ability^9^ and thus, crossing the Alps and entering Central Europe would not be possible^1,61^. However, there have been several records of *Phlebotomus* species north of the Alps^1^. On the one hand, this could indicate a natural dispersal towards northern area. On the other hand, their establishment in this region may have gone unnoticed or recorded specimen could have been recently introduced by e.g. soil or plant transports. An underestimation of occurrence for all *Phlebotomus* species due to their small body size may have potentially led to a sampling bias and must thus be kept in mind when interpreting the modeling results. In terms of dispersal ability, increasing tourism and global trade, which are assumed to support the dispersal of vectors, could also compensate for the limited dispersal ability of sandfly species in the future. In addition, anthropogenic impacts like pet dog travel, promote the carry-over and spread of phlebotomine vectors, but also of the *Leishmania* parasites^1,25^. Moreover, river valleys, such as those along the river Rhone in Southern France, could be regarded as migration corridors^42^. For projections of future climatic suitability, we assumed full-dispersal of the species, i.e. no limitations in dispersal ability and thus, the ability to reach every habitat climatically fitting. The area projected to be climatically suitable under future conditions but not under current conditions (future new range) might be much smaller than suggested by the climatic habitat suitability models, thus, future approaches should account for dispersal ability^42,62^ to further pinpoint areas of potential establishment.

An increased number of vector species as projected in this study, or a higher abundance of single species, especially species with a high vector competence, might result in a higher leishmaniasis transmission risk in Central and Northern Europe. However, if a species with low vector competence outcompetes another species with high vector competence (see discussion above on *P*. *perniciosus* and *P*. *ariasi*), the transmission risk for leishmaniasis could be reduced. Although the establishment of sandflies as the only known vector species for leishmaniasis^38^ is a necessary prerequisite, the establishment of vector species in an area alone does not inevitably lead to outbreaks of leishmaniasis. The main threat to human and dog health comes with the further introduction of the parasite species e.g. *Leishmania infantum* and *Leishmania tropica*, which are already established in Southern Europe^7^. Increased tourism and the trade and transport of numerous infected stray dogs by tourists or animal welfare organizations promotes the introduction of these parasites from the Mediterranean to other countries in Europe^8,25,63,64^. Pet owners and animal welfare organizations should therefore be informed about the risk of the disease, especially before travelling to or importing animals from endemic areas of leishmaniasis to assist in reducing the spread of the disease. Moreover, as already suggested by Menn *et al*.^25^, estimating the local risk in popular holiday locations in Southern Europe as well as a European recording system of the disease would be advantageous. Another important aspect of the habitat requirements of sandflies is the presence of suitable host species. Apart from dogs, several cosmopolitan wild rodent species and red foxes might serve as potential reservoir hosts. They are widely distributed in Europe and do not only occur in the same climatic habitats as sandflies, but also survive the non-active or low activity period of sandflies^29^. Hence, these rodents could presumably also play an important role in the transmission of leishmaniasis in Europe^29,65^. Moreover, pathogens require certain temperatures for a sufficient replication rate as well as for the infectious life stage^23,28^. The risk for humans to acquire leishmaniasis might currently be low, however, our models show that the climatic development could promote the expansion of sandfly vector species to non-endemic areas and therefore increase the risk of leishmaniasis outbreaks in Europe. Despite our efforts to incorporate climate projection variability by using different RCPs, we believe that in the future projections of climatic habitat suitability should be based on a larger number of GCMs seeing as this will help improve estimations of potential trajectories and better account for uncertainties.

## Material and Methods

### Occurrence data

For our models, we used occurrence data primarily based on data provided by Artemiev and Neronov^66^. Additional occurrence records were obtained from an intensive literature search to include also recent records. In total, 2236 data points were found in the literature (see also Fig. 1 for single species records). However, less occurrence records were considered for modeling (Table 1, Figs 2–5) as we used a grid with a spatial extent of the study area (latitude of 34°N – 72°N and longitude of 12°W–68°E) and a resolution of 5 arc minutes (~10 km) and associated the occurrences of each species to the center point of the respective grid cell to obtain only one occurrence point per grid cell matching the resolution of the climatic variable.

**Table 1:** Model specifications.

Species	Occurrence records	AUC ensemble models	Binary model threshold
*Phlebotomus alexandri*	68	0.986	42.65
*Phlebotomus ariasi*	126	0.992	53.8
*Phlebotomus mascittii*	71	0.994	61.15
*Phlebotomus neglectus*	113	0.986	49.55
*Phlebotomus papatasi*	385	0.955	65.05
*Phlebotomus perfiliewi*	168	0.989	48.15
*Phlebotomus perniciosus*	299	0.985	57.55
*Phlebotomus sergenti*	208	0.976	63.95
*Phlebotomus similis*	31	1	58.9
*Phlebotomus tobbi*	116	0.986	50.45
**Total**	**1585**		

Occurrence points used for modeling, modeling evaluation (AUC) and binary model thresholds [%].

### Climate data

We used bioclimatic variables provided by worldclim as explanatory variables^67,68^. The current climatic conditions refer to empirically collected data for the period 1960–1990. Out of the nineteen available bioclimatic variables we chose a subset of six variables for modeling. This subset comprises annual extrema (minimum and maximum) as well as a variable describing the annual variability for both, temperature and precipitation. Temperature and precipitation are relevant drivers for the distribution of sandflies^23,30–33^ (see also Introduction). More specifically, low temperature in winter^32^ and insufficient moisture^30,31^ are known to inhibit sandflies. Apart from ecological relevance, we avoided strong co-linearity of predictor variables and omitted one of the pair of variables from the model when Spearman rank correlation exceeded an absolute value of 0.75. As final variables, we included mean temperature of the warmest quarter (BIO10), mean temperature of the coldest  quarter (BIO11) and annual temperature range (BIO7). As precipitation variables we considered precipitation of the warmest quarter (BIO18), precipitation of the coldest quarter (BIO19) and precipitation seasonality (BIO15).

The climate data provided by worldclim were used for model training as well as for projections of climate habitat suitability under current climatic conditions. For projections of climate habitat suitability under future climatic conditions we used data from the Intergovernmental Panel on Climate Change^46^ (IPCC) 5th Assessment Report (AR5) provided by the International Centre for Tropical Agriculture (CIAT) and the CGIAR Research Program on Climate Change, Agriculture and Food Security (CCAFS)^69^. Data were available for three time periods: 2021–2040, 2041–2060 and 2061–2080. These scenarios assume different climatic futures based on greenhouse gas emissions projections and hence, the associated radiative forcing until 2100. RCP scenario 2.6 assumes only a low increase of radiative forcing and the temperature increase is expected to stay below the 2 °C threshold. RCP scenario 8.5 assumes 8.5 W/m² of radiative forcing and temperatures are supposed to range between 3.5–4.5 °C. For the other two RCP scenarios, 4.5 and 6.0, a medium increase of the radiative forcing is expected and future temperatures would vary between 2–4.5 °C^70,71^. Future models were built using only 2061–2080 RCP 8.5 scenario, except for two species (see further below).

For reasons of feasibility we only included one global circulation model (GCM) in our analysis. As there are no evaluation criteria for GCMs we decided to use one approach that is well-established, the global circulation model CSIRO-Mk3.6.0^72^, which has been used in other niche modeling studies before^51,73,74^.

### Ensemble forecasting

To model the climatic suitability for ten different sandfly species in Europe, an ensemble forecasting approach based on ten different algorithms was used. Consensus maps were built, considering the overall mean derived from all ten models with an AUC value > 0.85, and weighted by the AUC^53^. These consensus maps yield a robust estimation of the species’ climatic suitability^43,53^. The ten modeling algorithms used were: ANN – artificial neuronal networks, CTA – classification tree analysis, FDA – flexible discriminant analysis, GAM – generalized additive models, GBM – generalized boosted models, GLM – generalized linear models, MARS – multivariate adaptive regression splines, MAXENT – maximum entropy approach, RF – random forest, and SRE – surface range envelope. Modeling was done in the R environment^75^ (version 3.3.1) using the biomod2 package^76^ (version 3.3–7). We used default settings for all modeling algorithms except for GLM and MAXENT. In the GLM algorithm, a polynomial term was used with a stepwise procedure using Akaike’s Information Criterion (AIC). In the MAXENT algorithm we only used linear, quadratic and product features and increased the number of iterations to 10 000 to ensure that the algorithm converges. We randomly chose 10 000 pseudo-absences as background data for all algorithms.

We considered AUC values and variable importance for each algorithm as well as for the ensemble forecasting model for all species. The relative importance (calculated in biomod2) of the six climatic variables was converted into rank scaled values and averaged over the ten considered algorithms. Finally, the averaged rank of variable importance averaged over the ten species was calculated to identify variables that shape the distribution of the considered species.

### Mapping of modeling results

All our maps were built using ESRI ArcGIS^77^ (Release 10.3). We displayed the projected climatic suitability for the ten sandfly species under current climatic conditions (Fig. 2) and under future climatic conditions considering only the time period 2061–2080 and the RCP 8.5 scenario (Fig. 3). In addition, we used *P*. *mascittii* and *P*. *perniciosus* as model examples to assess the variability of our results due to different RCP scenarios (Fig. 4). These two species were chosen as model examples; *P*. *perniciosus* is assumed to be the main vector for leishmaniasis in the Mediterranean area^78,79^ and *P*. *mascittii* is the most northern distributed *Phlebotomus* species in Europe^27^. Thus, one species represents the outermost populations of the European sandflies and the other one is a well-known, risk-associated species already^36^.

Applying the threshold that minimizes the difference between sensitivity and specificity (also referred to as “equal training sensitivity and specificity threshold rule”), the continuous climatic suitability maps were converted into binary maps for all considered sandfly species (for threshold values see Table 1). Based on these binary modeling results we created maps reflecting changes in climatic suitability and identifying regions with stable climatic suitability, possible extinction areas and areas of potential new establishments (Fig. 5). We defined areas with climatic habitat suitability under current climate conditions but no climatic suitability under future climate conditions as extinction areas. Areas that provide only climatically suitable habitats in the future but not under current climate were considered as areas of potential range expansion, and regions where species will find adequate climatic conditions under current and future climate were defined as areas of stable climatic suitability. Comparisons were made between current climatic suitability and the future model for the time period 2061–2080 and RCP scenario 8.5.

Based on the binary modeling results, diversity maps were generated (Fig. 6). These maps show the number of sandfly species for which suitable climatic conditions exist within the respective pixel. They were built for all considered time periods (2021–2040, 2041–2060, 2061–2080) and all four RCP scenarios (RCP 2.6, RCP 4.5, RCP 6.0, RCP 8.5).

## Electronic supplementary material

Supplementary material
