# Supplementary material for: Modeling the climatic suitability of leishmaniasis vector species in Europe
Source: Sci Rep. 2017 Oct 17;7:13325. doi: 10.1038/s41598-017-13822-1 (PMC5645347; doi:10.1038/s41598-017-13822-1)
Supplement: Supplementary file 1 — Supplementary material [file 41598_2017_13822_MOESM1_ESM.pdf]

Title

Modeling the climatic suitability of leishmaniasis vector species in Europe

Authors

Lisa K. Koch[1,2,*], Judith Kochmann[1,2], Sven Klimpel[1,2], Sarah Cunze[1,2]

Affiliation

[1]Goethe-University, Institute of Ecology, Evolution and Diversity, Frankfurt/ Main, D-60438, Germany
[2]Senckenberg Gesellschaft für Naturforschung, Senckenberg Biodiversity and Climate Research Centre, Frankfurt/ Main, D-60325, Germany

*Corresponding author: Lisa K. Koch (Email: Lisa.Koch@senckenberg.de)

Abbreviations:

Modeling Algorithms

ANN: artificial neuronal networks

CTA: classification tree analysis

FDA: flexible discriminant analysis

GAM: generalized additive models

GBM: generalized boosted models

GLM: generalized linear models

MARS: multivariate adaptive regression splines

MAXENT: maximum entropy approach

RF: random forest

SRE: surface range envelope

Bioclimatic variables

BIO7: annual temperature range

BIO10: mean temperature of the warmest quarter

BIO11: mean temperature of the warmest quarter

BIO15: precipitation seasonality

BIO18: precipitation of the warmest quarter

BIO19: precipitation of the coldest quarter

Supplementary Table S1. AUC values (calculated using the R[1] package biomod2[2]). Results are given for each of the 10 *Phlebotomus* species and are shown for each model and the ensemble model (EM).

	ANN	CTA	FDA	GAM	GBM	GLM	MAXENT	MARS	RF	SRE	EM
Phlebotomus alexandri	0.926	0.882	0.914	0.958	0.97	0.94	0.945	0.943	1	0.841	0.986
Phlebotomus ariasi	0.956	0.942	0.951	0.973	0.971	0.96	0.973	0.963	1	0.818	0.992
Phlebotomus mascittii	0.954	0.948	0.934	0.978	0.981	0.953	0.956	0.956	1	0.854	0.994
Phlebotomus neglectus	0.949	0.93	0.921	0.955	0.969	0.938	0.963	0.945	1	0.81	0.986
Phlebotomus papatasi	0.886	0.867	0.884	0.894	0.922	0.886	0.906	0.891	1	0.749	0.955
Phlebotomus perfiliewi	0.963	0.913	0.941	0.965	0.972	0.956	0.974	0.96	1	0.852	0.989
Phlebotomus perniciosus	0.947	0.931	0.948	0.957	0.966	0.95	0.965	0.951	1	0.841	0.985
Phlebotomus sergenti	0.928	0.889	0.91	0.921	0.937	0.91	0.929	0.915	1	0.778	0.976
Phlebotomus similis	0.961	0.927	0.942	0.933	0.991	0.834	0.954	0.977	1	0.831	1
Phlebotomus tobbi	0.963	0.909	0.894	0.959	0.973	0.934	0.957	0.945	1	0.813	0.986

Supplementary Table S2. Relative variable importance (calculated using the R[1] package biomod2[2]). Results are given for each of the 10 *Phlebotomus* species and for each model. For abbreviations of climatic variables (BIO xy, left row) and model type (upper row) refer to Material and Methods.

Phlebotomus alexandri

	ANN	CTA	FDA	GAM	GBM	GLM	MAXENT	MARS	RF	SRE
BIO7	0.109	0	0	0.333	0.059	0.12	0.02	0.053	0.23	0.266
BIO10	0.057	0	0.161	0.164	0.011	0.146	0.03	0	0.382	0.096
BIO11	0.385	0	0	0.542	0.037	0.622	0.152	0.184	0.314	0.241
BIO15	0.265	0	0.724	0.221	0.167	0.026	0.136	0.127	0.257	0.32
BIO18	0.495	0.986	0	0.657	0.805	0.79	0.702	0.597	0.581	0.704
BIO19	0.234	0	0	0.135	0.046	0.007	0.006	0.033	0.138	0.077

Phlebotomus ariasi

	ANN	CTA	FDA	GAM	GBM	GLM	MAXENT	MARS	RF	SRE
BIO7	0.48	0.126	0.392	0.579	0.169	0.4	0.221	0.397	0.264	0.577
BIO10	0.577	0.118	0.476	0.461	0.077	0.593	0.378	0.56	0.418	0.112
BIO11	1	0.962	1	0.975	0.696	1	0.978	1	0.646	0.538
BIO15	0.314	0.069	0.089	0.236	0.055	0.273	0.203	0.238	0.142	0.086
BIO18	0.195	0.233	0	0.26	0.095	0.246	0.272	0.13	0.416	0.128
BIO19	0.234	0.024	0	0.175	0.031	0.104	0.055	0.061	0.279	0.176

Phlebotomus mascittii

	ANN	CTA	FDA	GAM	GBM	GLM	MAXENT	MARS	RF	SRE
BIO7	0.567	0.883	0.165	0.653	0.534	0.552	0.45	0.582	0.5	0.568
BIO10	0.3	0.311	0.107	0.528	0.343	0.388	0.241	0.382	0.146	0.266
BIO11	0.648	0	0.486	0.428	0.363	0.313	0.482	0.04	0.244	0.462
BIO15	0.147	0	0	0.209	0.001	0.12	0.08	0	0.107	0.081
BIO18	0.468	0	0.215	0.29	0.034	0.17	0.244	0.164	0.351	0.14
BIO19	0.349	0	0.103	0.412	0.273	0.261	0.146	0.265	0.41	0.435

Phlebotomus neglectus

	ANN	CTA	FDA	GAM	GBM	GLM	MAXENT	MARS	RF	SRE
BIO7	0.978	0.355	0.667	0.686	0.347	0.651	0.654	0.744	0.431	0.446
BIO10	0.999	0.306	0.571	0.638	0.202	0.586	0.532	0.589	0.434	0.182
BIO11	0.617	0.603	0.298	0.589	0.354	0.397	0.146	0.395	0.34	0.55
BIO15	0.308	0	0	0.143	0.027	0.033	0.186	0.183	0.3	0.061
BIO18	0.305	0	0.057	0.328	0.027	0.11	0.285	0.351	0.177	0.099
BIO19	0.511	0.321	0.752	0.603	0.538	0.507	0.662	0.494	0.609	0.319

Phlebotomus papatasi

	ANN	CTA	FDA	GAM	GBM	GLM	MAXENT	MARS	RF	SRE
BIO7	0.088	0.04	0.068	0.338	0.024	0.373	0.106	0.362	0.248	0.195
BIO10	0.168	0.47	0.193	0.109	0.245	0.076	0.066	0.084	0.451	0.303
BIO11	0.695	0.408	0.534	1	0.499	1	0.87	1	0.477	0.557
BIO15	0.204	0	0.287	0.091	0.011	0.098	0.235	0.173	0.255	0.09
BIO18	0.14	0.164	0.052	0.047	0.028	0.058	0.08	0.064	0.359	0.115
BIO19	0.143	0.03	0	0.002	0.012	0.004	0.023	0	0.216	0.066

Phlebotomus perfiliewi

	ANN	CTA	FDA	GAM	GBM	GLM	MAXENT	MARS	RF	SRE
BIO7	0.594	0.48	0.059	0.604	0.445	0.589	0.472	0.5	0.269	0.5
BIO10	0.418	0.422	0.195	0.613	0.326	0.649	0.425	0.465	0.484	0.349
BIO11	0.854	0.504	0.379	0.492	0.446	0.334	0.099	0.234	0.318	0.478
BIO15	0.169	0	0.052	0.09	0.035	0.014	0.088	0.059	0.195	0.08
BIO18	0.295	0	0.335	0.143	0.12	0.14	0.18	0.114	0.252	0.326
BIO19	0.14	0.223	0.26	0.369	0.217	0.302	0.222	0.249	0.395	0.305

Phlebotomus perniciosus

	ANN	CTA	FDA	GAM	GBM	GLM	MAXENT	MARS	RF	SRE
BIO7	0.172	0.381	0.133	0.383	0.344	0.255	0.329	0.127	0.311	0.493
BIO10	0.283	0.315	0.044	0.157	0.259	0.139	0.07	0.129	0.256	0.221
BIO11	0.985	0.659	0.593	0.893	0.668	1	0.739	0.991	0.464	0.606
BIO15	0.143	0.09	0.041	0.047	0.044	0.078	0.049	0.072	0.123	0.07
BIO18	0.225	0	0	0.039	0.002	0.019	0.036	0	0.141	0.112
BIO19	0.217	0.035	0.028	0.092	0.041	0.065	0.059	0.033	0.247	0.186

Phlebotomus sergenti

	ANN	CTA	FDA	GAM	GBM	GLM	MAXENT	MARS	RF	SRE
BIO7	0.289	0	0.268	0.285	0.004	0.453	0.011	0.404	0.218	0.135
BIO10	0.254	0	0.097	0.142	0.019	0.177	0.052	0.161	0.354	0.079
BIO11	0.625	0.134	0.805	0.944	0.091	1	0.734	1	0.402	0.392
BIO15	0.232	0.581	0.409	0.594	0.444	0.572	0.583	0.686	0.669	0.656
BIO18	0.365	0.288	0.037	0.068	0.139	0.011	0.077	0.034	0.469	0.167
BIO19	0.137	0	0.014	0.021	0.022	0.019	0.08	0.014	0.149	0.089

Phlebotomus similis

	ANN	CTA	FDA	GAM	GBM	GLM	MAXENT	MARS	RF	SRE
BIO7	0.747	0.636	0.823	0.737	0.45	0.564	0.328	0.347	0.465	0.382
BIO10	0.608	0.357	0.645	0.508	0.531	0.577	0.377	0.509	0.232	0.523
BIO11	0.511	0.363	0.387	0.669	0.463	0	0.028	0.445	0.334	0.376
BIO15	0.363	0.31	0.088	0.424	0.147	0.047	0.165	0.138	0.465	0.053
BIO18	0.621	0.248	0.068	0.469	0.398	0.161	0.29	0.303	0.113	0.366
BIO19	0.418	0.421	0.48	0.46	0.423	0	0.251	0.449	0.278	0.355

Phlebotomus tobbi

	ANN	CTA	FDA	GAM	GBM	GLM	MAXENT	MARS	RF	SRE
BIO7	0.551	0	0.216	0.401	0.137	0.301	0.171	0.296	0.28	0.3
BIO10	0.39	0	0.174	0.23	0.042	0.119	0.1	0.133	0.265	0.321
BIO11	0.911	0.914	0.63	0.837	0.614	0.997	0.409	0.989	0.377	0.419
BIO15	0.089	0.223	0.332	0.237	0.165	0.263	0.393	0.223	0.124	0.144
BIO18	0.408	0.094	0.328	0.263	0.06	0.171	0.326	0.103	0.473	0.353
BIO19	0.378	0	0.288	0.1	0.07	0.04	0.09	0.12	0.253	0.139

Supplementary Table S3. Ranked variable importance. Results are given for each of the 10 *Phlebotomus* species and are shown for single models and for the mean over all 10 algorithms. 1 = most important variable, 6 = least important variable.

Phlebotomus alexandri

	ANN	CTA	FDA	GAM	GBM	GLM	MAXENT	MARS	RF	SRE	Mean
BIO7	5	2	3	3	3	4	5	4	5	3	3.7
BIO10	6	2	2	5	6	3	4	6	2	5	4.1
BIO11	2	2	3	2	5	2	2	2	3	4	2.7
BIO15	3	2	1	4	2	5	3	3	4	2	2.9
BIO18	1	1	3	1	1	1	1	1	1	1	1.2
BIO19	4	2	3	6	4	6	6	5	6	6	4.8

Phlebotomus ariasi

	ANN	CTA	FDA	GAM	GBM	GLM	MAXENT	MARS	RF	SRE	Mean
BIO7	3	3	3	2	2	3	4	3	5	1	2.9
BIO10	2	4	2	3	4	2	2	2	2	5	2.8
BIO11	1	1	1	1	1	1	1	1	1	2	1.1
BIO15	4	5	4	5	5	4	5	4	6	6	4.8
BIO18	6	2	5	4	3	5	3	5	3	4	4
BIO19	5	6	5	6	6	6	6	6	4	3	5.3

Phlebotomus mascittii

	ANN	CTA	FDA	GAM	GBM	GLM	MAXENT	MARS	RF	SRE	Mean
BIO7	2	1	3	1	1	1	2	1	1	1	1.4
BIO10	5	2	4	2	3	2	4	2	5	4	3.3
BIO11	1	3	1	3	2	3	1	5	4	2	2.5
BIO15	6	3	6	6	6	6	6	6	6	6	5.7
BIO18	3	3	2	5	5	5	3	4	3	5	3.8
BIO19	4	3	5	4	4	4	5	3	2	3	3.7

Phlebotomus neglectus

	ANN	CTA	FDA	GAM	GBM	GLM	MAXENT	MARS	RF	SRE	Mean
BIO7	2	2	2	1	3	1	2	1	3	2	1.9
BIO10	1	4	3	2	4	2	3	2	2	4	2.7
BIO11	3	1	4	4	2	4	6	4	4	1	3.3
BIO15	5	5	6	6	5	6	5	6	5	6	5.5
BIO18	6	5	5	5	5	5	4	5	6	5	5.1
BIO19	4	3	1	3	1	3	1	3	1	3	2.3

Phlebotomus papatasi

	ANN	CTA	FDA	GAM	GBM	GLM	MAXENT	MARS	RF	SRE	Mean
BIO7	6	4	4	2	4	2	3	2	5	3	3.5
BIO10	3	1	3	3	2	4	5	4	2	2	2.9
BIO11	1	2	1	1	1	1	1	1	1	1	1.1
BIO15	2	6	2	4	6	3	2	3	4	5	3.7
BIO18	5	3	5	5	3	5	4	5	3	4	4.2
BIO19	4	5	6	6	5	6	6	6	6	6	5.6

Phlebotomus perfiliewi

	ANN	CTA	FDA	GAM	GBM	GLM	MAXENT	MARS	RF	SRE	Mean
BIO7	2	2	5	2	2	2	1	1	4	1	2.2
BIO10	3	3	4	1	3	1	2	2	1	3	2.3
BIO11	1	1	1	3	1	3	5	4	3	2	2.4
BIO15	5	5	6	6	6	6	6	6	6	6	5.8
BIO18	4	5	2	5	5	5	4	5	5	4	4.4
BIO19	6	4	3	4	4	4	3	3	2	5	3.8

Phlebotomus perniciosus

	ANN	CTA	FDA	GAM	GBM	GLM	MAXENT	MARS	RF	SRE	Mean
BIO7	5	2	2	2	2	2	2	3	2	2	2.4
BIO10	2	3	3	3	3	3	3	2	3	3	2.8
BIO11	1	1	1	1	1	1	1	1	1	1	1
BIO15	6	4	4	5	4	4	5	4	6	6	4.8
BIO18	3	6	6	6	6	6	6	6	5	5	5.5
BIO19	4	5	5	4	5	5	4	5	4	4	4.5

Phlebotomus sergenti

	ANN	CTA	FDA	GAM	GBM	GLM	MAXENT	MARS	RF	SRE	Mean
BIO7	3	4	3	3	6	3	6	3	5	4	4
BIO10	4	4	4	4	5	4	5	4	4	6	4.4
BIO11	1	3	1	1	3	1	1	1	3	2	1.7
BIO15	5	1	2	2	1	2	2	2	1	1	1.9
BIO18	2	2	5	5	2	6	4	5	2	3	3.6
BIO19	6	4	6	6	4	5	3	6	6	5	5.1

Phlebotomus similis

	ANN	CTA	FDA	GAM	GBM	GLM	MAXENT	MARS	RF	SRE	Mean
BIO7	1	1	1	1	3	2	2	4	1	2	1.8
BIO10	3	4	2	3	1	1	1	1	5	1	2.2
BIO11	4	3	4	2	2	5	6	3	3	3	3.5
BIO15	6	5	5	6	6	4	5	6	1	6	5
BIO18	2	6	6	4	5	3	3	5	6	4	4.4
BIO19	5	2	3	5	4	5	4	2	4	5	3.9

Phlebotomus tobbi

	ANN	CTA	FDA	GAM	GBM	GLM	MAXENT	MARS	RF	SRE	Mean
BIO7	2	4	5	2	3	2	4	2	3	4	3.1
BIO10	4	4	6	5	6	5	5	4	4	3	4.6
BIO11	1	1	1	1	1	1	1	1	2	1	1.1
BIO15	6	2	2	4	2	3	2	3	6	5	3.5
BIO18	3	3	3	3	5	4	3	6	1	2	3.3
BIO19	5	4	4	6	4	6	6	5	5	6	5.1

Supplementary Table S4. Averaged ranked variable importance. Results for each variable are given as averages over all 10 algorithms (models) and are shown for each of the 10 *Phlebotomus* species and for the mean of 10 species. 1 = most important variable, 6 = least important variable.

	bio07	bio10	bio11	bio15	bio18	bio19
Phlebotomus alexandri	3.7	4.1	2.7	2.9	1.2	4.8
Phlebotomus ariasi	2.9	2.8	1.1	4.8	4	5.3
Phlebotomus mascittii	1.4	3.3	2.5	5.7	3.8	3.7
Phlebotomus neglectus	1.9	2.7	3.3	5.5	5.1	2.3
Phlebotomus papatasi	3.5	2.9	1.1	3.7	4.2	5.6
Phlebotomus perfiliewi	2.2	2.3	2.4	5.8	4.4	3.8
Phlebotomus perniciosus	2.4	2.8	1	4.8	5.5	4.5
Phlebotomus sergenti	4	4.4	1.7	1.9	3.6	5.1
Phlebotomus similis	1.8	2.2	3.5	5	4.4	3.9
Phlebotomus tobbi	3.1	4.6	1.1	3.5	3.3	5.1
**Mean**	2.69	3.21	2.04	4.36	3.95	4.41

References:

1. R Core Team. R: A language and environment for statistical computing. R Foundation for Statistical ComputingVienna, Austria. URL https://www.R-project.org/. Version R version 3.3.1 (2016-06-21). (2016).
2. Thuiller, W., Georges, D., Engler, R. & Breiner, F. biomod2: Ensemble Platform for Species Distribution Modeling. R package version 3.3-7. https://CRAN.R-project.org/package=biomod2. (2016).
